# Neural circuits expressing the serotonin 2C receptor regulate memory in mice and humans

**DOI:** 10.1002/alz.095039

**Published:** 2025-01-09

**Authors:** Hesong Liu, Brouwers Bas, Yang He, Hailan Liu, Na Yin, yongjie Yang, Meng Yu, Chunmei Wang, Yong Xu

**Affiliations:** ^1^ Baylor College of Medicine, Houston, TX USA; ^2^ University of Cambridge, Cambridge, Cambridge United Kingdom

## Abstract

**Background:**

A decline in memory is a hallmark of Alzheimer’s disease (AD). Experiments in rodents and post‐mortem studies in humans with AD suggest that serotonin (5‐HT) plays a role in memory, but the molecular and neural mechanisms mediating its effects are unknown.

**Method:**

100,000 individuals in UK Biobank were studied for the role of serotonin 2C receptor (5‐HT_2C_R) in the regulation of memory. We examined both wild‐type (WT) and transgenic mice (F327L) expressing a human loss‐of‐function serotonin 2C receptor (*HTR2C*). Memory function was assessed using Radial Arm Water Maze test. Additionally, we conducted neural tracing by injecting retrograde viruses expressing GFP into the vCA1 of WT mice. We disrupted serotonin synthesis in vCA1‐projecting neurons using AAV‐Cre virus in *Tph2‐CreER* mice and deleted 5‐HT_2C_Rs on vCA1 neurons in *Htr2c^flox/Y^* mice. Furthermore, we evaluated the integrity of 5‐HT to vCA1 circuit using *Tph2‐CreER/Rosa26‐LSL‐tdTOMATO/ App^NL‐G‐F^* mice. Lastly, we explored the effects of lorcaserin, a selective 5‐HT_2C_R agonist, in *App^NL‐G‐F^* mice.

**Result:**

Our findings revealed that a rare loss‐of‐function mutation in *HTR2C* significantly impaired short‐term memory in both humans and F327L mice. Neural tracing experiments demonstrated that midbrain serotonin neurons project to the vCA1 hippocampal region. Disruption of serotonin synthesis in vCA1‐projecting neurons and deletion of 5‐HT_2C_Rs on vCA1 neurons led to memory impairment. Notably, the 5‐HT to vCA1 circuit was disrupted in *App^NL‐G‐F^* mice. However, lorcaserin corrected memory loss in a mouse model of amyloid pathology.

**Conclusion:**

The 5‐HT to vCA1 circuit regulates working memory. By utilizing 5‐HT_2C_R agonists, we can potentially bypass the damages in this circuit and enhance memory function in *App^NL‐G‐F^* mice.

